# Amelioration of Cold Injury-Induced Cortical Brain Edema Formation by Selective Endothelin ET_B_ Receptor Antagonists in Mice

**DOI:** 10.1371/journal.pone.0102009

**Published:** 2014-07-07

**Authors:** Shotaro Michinaga, Marina Nagase, Emi Matsuyama, Daisuke Yamanaka, Naoki Seno, Mayu Fuka, Yui Yamamoto, Yutaka Koyama

**Affiliations:** Laboratory of Pharmacology, Faculty of Pharmacy, Osaka Ohtani University, Tonda-bayashi, Osaka, Japan; Biological Research Centre of the Hungarian Academy of Sciences, Hungary

## Abstract

Brain edema is a potentially fatal pathological condition that often occurs in stroke and head trauma. Following brain insults, endothelins (ETs) are increased and promote several pathophysiological responses. This study examined the effects of ET_B_ antagonists on brain edema formation and disruption of the blood-brain barrier in a mouse cold injury model (Five- to six-week-old male ddY mice). Cold injury increased the water content of the injured cerebrum, and promoted extravasation of both Evans blue and endogenous albumin. In the injury area, expression of prepro-ET-1 mRNA and ET-1 peptide increased. Intracerebroventricular (ICV) administration of BQ788 (ET_B_ antagonist), IRL-2500 (ET_B_ antagonist), or FR139317 (ET_A_ antagonist) prior to cold injury significantly attenuated the increase in brain water content. Bolus administration of BQ788, IRL-2500, or FR139317 also inhibited the cold injury-induced extravasation of Evans blue and albumin. Repeated administration of BQ788 and IRL-2500 beginning at 24 h after cold injury attenuated both the increase in brain water content and extravasation of markers. In contrast, FR139317 had no effect on edema formation when administrated after cold injury. Cold injury stimulated induction of glial fibrillary acidic protein-positive reactive astrocytes in the injured cerebrum. Induction of reactive astrocytes after cold injury was attenuated by ICV administration of BQ788 or IRL-2500. These results suggest that ET_B_ receptor antagonists may be an effective approach to ameliorate brain edema formation following brain insults.

## Introduction

After the onset of stroke and head trauma, brain edema often occurs. Brain edema is a potentially fatal pathological state where accumulation of brain water impairs nerve function owing to elevation of intracranial pressure. The pathogenesis of brain edema is classified into two types: vasogenic edema and cytotoxic edema [1], [2]. Vasogenic edema is accompanied by disruption of the blood-brain barrier (BBB) and is mainly involved in the elevation of intracranial pressure. Disruption of the BBB allows extravasation of blood proteins into brain parenchyma and water accumulation in damaged areas, which underlies the pathogenesis of vasogenic edema [2], [3]. Despite the seriousness of the condition, medications to prevent edema formation have not been established, and only symptomatic treatments that assist in the removal of excess water from edematous brain are currently available [4], [5]. Astrocytes have been proposed to play a role in the disruption of BBB after brain insults. In brain, astrocytes are in close contact with vascular endothelial cells via the end-feet of their processes. Because this anatomical structure creates restricted permeability to blood components, a large part of the BBB function is due to the interactions between astrocytes and vascular endothelial cells. Permeability of brain microvessels is not static, but is dynamically regulated by astrocyte-derived factors [6], [7]. Following brain insults, astrocytes change their phenotype to become reactive astrocytes, which are characterized by hypertrophy and expression of glial fibrillary acidic protein (GFAP). Reactive astrocytes release many soluble factors, including growth factors, cytokines, chemokines, and vascular permeability factors, which modulate the pathophysiological responses of other cells around the injured area [8], [9]. Along with the conversion to reactive astrocytes, production of astrocyte-derived vascular permeability factors such as matrixmetalloproteinases (MMPs) and vascular endothelial growth factor (VEGF) is increased, and these factors induce disruption of BBB and vasogenic edema [10], [11], [12], [13], [14]. Thus, reduction of astrocytic activation after brain insults is expected to be a novel therapeutic strategy to prevent vasogenic edema formation [15].

Production of endothelins (ETs), a family of vasoconstricting peptides, is increased in damaged nerve tissues [16], [17]. ETs have two distinct types of receptors, ET_A_ and ET_B_ receptors, and the cellular distributions of ET_A_ and ET_B_ receptors in brain are different. ET_A_ receptors are present in vascular smooth muscle and are involved in the aggravation of ischemic brain damage by inducing vasospasm of brain arteries [18]. ET_B_ receptors are predominantly expressed in astrocytes [19], [20], [21]. Administration of a selective ET_B_ agonist into rat brain increased the numbers of GFAP-positive reactive astrocytes [22], [23]. Induction of reactive astrocytes by brain ischemia and traumatic injury was reduced by an ET_B_ antagonist [24], [25]. These findings suggest that activation of astrocytic ET_B_ receptors promotes the phenotypic conversion to reactive astrocytes. While the pharmacological significance of ET_B_ receptors in modulating astrocytic function in damaged nerve tissues has been shown [26], the effects of ET_B_ antagonists on vasogenic edema and disruption of BBB have rarely been examined.

Focal cold injury is used to model vasogenic brain edema in rodents, because freezing and thawing of nerve tissues directly impairs the integrity of vascular endothelial cells and enhances extravasation of blood proteins through disrupted BBB [27], [28], [29]. In this study, the effects of ET_B_ antagonists on brain edema formation and disruption of BBB were examined in a mouse cortical cold injury model. We found that administration of the selective ET_B_ antagonists BQ788 and IRL-2500 prior to cold injury attenuated development of brain edema and disruption of BBB. Furthermore, the inhibitory actions of ET_B_ antagonists were also observed when they were administered after cold injury-induced brain edema had developed.

**Figure 1:**
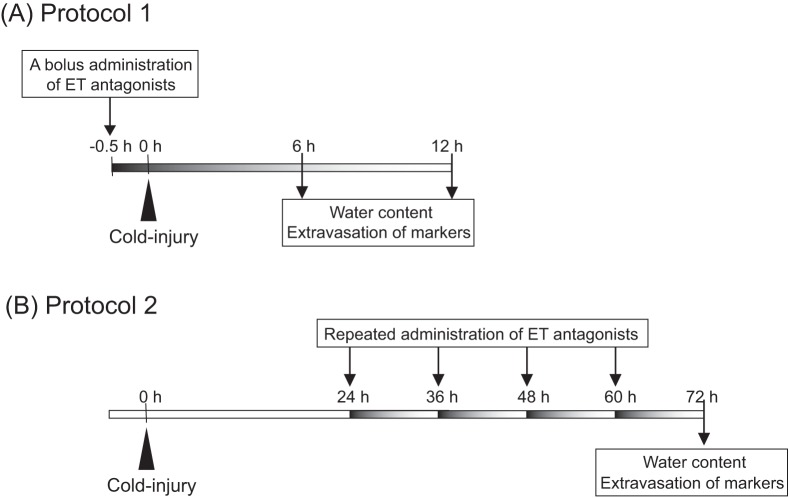
Experimental schedules for ICV administration of ET receptor antagonists. **(A) Protocol 1:** Administration prior to cold injury. A bolus injection of ET antagonist into the right cerebroventricle was made 30(arrow). Brain water content and extravasation of markers were measured at 6 h and 12 h after cold injury. **(B) Protocol 2:** Repeated ICV administration after cold injury. ET antagonists were repeatedly administered at 12-h intervals starting from 24 h after cold injury (arrows). Brain water content and extravasation of markers were measured at 72 h after cold injury.

## Materials and Methods

### Focal cold injury to mouse cerebrum

All experimental protocols conformed to the National Institutes of Health guiding principles for the care and use of animals (NIH Publications No. 8023), and all experiments were approved by the Animal Experiment Committee of Osaka Ohtani University (No. 0605). In this study, all efforts were made to minimize animal suffering and to reduce the number of animals used. All experiments used animals were performed in animal experiment room in Osaka Ohtani University. Five- to six-week-old male ddY mice (SLC, Shizuoka, Japan) were housed in pathogen free conditions and provided with a commercial diet and water ad libitum under controlled conditions (temperature: 23±2°C, humidity: 55±5% and a 12 h light/dark cycle). The weight of mice was within defined areas of 27–32 g before ICV administration and cold injury. Mice were anesthetized by pentobarbital (i.p. 50 mg/kg), and placed in a stereotactic device. The scalp was incised at the midline and the skull was exposed. To induce cold injury in mouse cerebrum, a copper rod (15-cm length, 5-mm diameter) was kept in a 50-mL conical tube filled with powdered dry ice. The cooled rod was set on a micromanipulator with the conical tube. The tip of the cooled rod was applied to the skull of the left hemisphere at a position of 3 mm posterior and 5 mm lateral to bregma for 60 s. Then, the incision was sutured and the mice were kept in raising cages. For sham-operated mice, a copper rod not cooled by dry ice was applied in a similar manner. The total number of mice in each experimental group was minimally determined for statistical significance and decrease of bias was within 30 to 42 animals. Because the effects of ET receptor antagonists on induction of reactive astrocytes and vascular permeability factors were preliminarily examined in cultured astrocytes, we could minimize animal suffering and the number of animals used in this study. Mice which did not show normal development of body weight, i.e., under 25.0 g at 6 week-old, were excluded from observations. The percentages of mice died or showing the reduced weight were less than 10% of total animals used, which did not differ among the experiment groups.

### Intracerebroventricular (ICV) administration of ET antagonists

In this study, the following ET receptor antagonists were used: BQ788 (ET_B_-selective; Phoenix Pharmaceuticals Inc., Burlingame, CA, USA), IRL-2500 (ET_B_-selective; Tocris Bioscience, Ellisville, MS, USA), FR139317 (ET_A_-selective; Funakoshi Co. Ltd., Osaka, Japan), and bosentan (nonspecific ET_A_- and ET_B_-receptor antagonist; Toronto Research Chemicals Inc., North York, Canada). To exclude possible influences on systemic circulation and differences in accessibility to the central nervous system, ET antagonists were directly injected into mouse cerebroventricles. The effects of ET antagonists on cold injury-induced edema formation were examined following two different protocols of ICV administration. Mice were divided into six groups for ICV administration: (1) sham group, (2) vehicle, (3) BQ788, (4) IRL-2500, (5) FR139317 and (6) bosentan. After cold injury, mice were randomly selected for ICV administration. The ICV administrations and experimental assessment were performed in order of vehicle, BQ788, IRL-2500, FR139117 and bosentan.

#### Protocol 1: Administration prior to cold injury (Fig. 1A)

To examine effects on the development of brain edema, ET antagonists were administered into the right cerebroventricle 30 min before cold injury. Mice were placed in a stereotactic device under pentobarbital anesthesia. The skull was exposed, and a burr hole was made with a dental drill at a position of 0.1 mm posterior and 1.0 mm right lateral to bregma. Stock solutions of ET antagonists, which were dissolved in dimethylsulfoxide (DMSO), were diluted with sterilized saline. Diluted drug solutions were loaded in a microsyringe (34 gauge; Hamilton, Reno, NV, USA) and the syringe tip was positioned at 2.5 mm below bregma through the burr hole. Two microliters of drug solution was injected into the right cerebroventricle at a rate of 1 µL/min. For controls, saline containing 5% DMSO was injected in a similar manner.

#### Protocol 2: Repeated ICV administration after cold injury (Fig. 1B)

To examine the effects on recovery in edematous brain, ET antagonists were repeatedly administered from 24 h after cold injury. Three days before cold injury, a guide cannula (CXG-X type; Eicom, Japan) was fixed to the skull (0.1 mm posterior, 1.0 mm right lateral to bregma). Repeated ICV administrations of ET antagonists were started at 24 h after cold injury was performed. Diluted solutions of ET antagonists in a microsyringe were repeatedly injected through the implanted guide cannula at 12-h intervals such as 9∶00 and 21∶00. Brain edema and extravasation of markers were measured at 72 h after cold injury. At that time, mice subjected to cold injury were randomly selected for each of ET antagonist administrations.

### Measurement of brain water content

Brain edema was evaluated by measurement of brain water content. At the times indicated after cold injury, the brain was removed under deep anesthesia. The brain was placed on a brain slicer (Muromachi, Tokyo, Japan) and a sagittal section (4 mm thick, between 1 and 5 mm posterior to bregma) was cut. The cerebrum was dissected from the slice and the tissue weight (wet weight) was measured. The cerebral tissue was incubated at 70°C for 12 h and the weight (dry weight) was measured. Percentage of water content was determined by the formula: water content (%)  =  (wet weight−dry weight)×100/wet weight. To confirm dose-dependent effects of ET antagonists, mice were divided into six groups for ICV administration: (1) sham group, (2) vehicle, ET antagonists at (3) 1 µg, (4) 5 µg, (5) 10 µg and (6) 50 µg.

### Measurement of endogenous serum albumin extravasation

To remove endogenous albumin in brain vessels, mice were perfused from the left cardiac ventricle with 50 mL of phosphate-buffered saline (PBS) at the indicated times after cold injury. The perfused brain was removed, and a sagittal section (4 mm thick) was cut as described above. The cerebrum was dissected from the slice and the tissue weight was measured. The tissue was homogenized in 200 µL of cell lysis buffer (20 mM tris/HCl, pH 7.4, 150 mM NaCl, 1% (v/v) NP-40, 0.5% (v/v) deoxycholic acid, 0.1% (w/v) SDS, 0.5% (w/v) EDTA, 2 mM phenylmethylsulfonyl fluoride, and 10 µg/mL aprotinin). The lysates were centrifuged at 15,000 g for 10 min. The amounts of mouse albumin in the supernatant were measured with an ELISA kit (mouse albumin ELISA Quantitation Set; Bethyl Laboratory Inc., Montgomery, MO, USA) according to the manufacturer’s protocol. Data are shown as calculated albumin content (ng) per weight of cerebral hemisphere (g).

### Extravasation of Evans blue from blood

Permeability of the BBB was evaluated by extravasation of Evans blue. Ninety minutes before determination of vascular permeability, 4% Evans blue in saline was injected into the tail vein at 3 mL/kg. After perfusion with 50 mL saline, the brain was removed, and a sagittal section (4 mm thick) was cut as described above. At the same time, an aliquot of mouse blood was collected for determination of serum Evans blue content. The cerebrum was dissected from the slice and the tissue weight was measured. The tissues were immersed in 400 µL formamide at 55°C overnight to extract the Evans blue. The amount of Evans blue in the extract was measured by absorbance at 655 nm. The percentage of Evans blue extravasation was determined by the formula Evans blue (%)  =  Evans blue in brain (µg/g)×100/Evans blue in serum (µg/mL).

### Measurement of prepro-ET-1 mRNA levels by quantitative RT-PCR

Total RNA from the cerebrum in sagittal brain sections was extracted using an acid-phenol method followed by repeated isopropanol precipitation. First-strand cDNA was synthesized from the total RNA as described previously [30]. Prepro-ET-1 mRNA levels in each sample were determined by real-time PCR using SYBR Green fluorescent probes (Toyobo, Tokyo, Japan). The following primer pairs were used: mouse prepro-ET-1, 5′- TGT GTC TAC TTC TGC CAC CT-3′ and 5′-CAC CAG CTG CTG ATA GAT AC-3′; mouse glyceraldehyde-3-phosphate dehydrogenase (G3PDH), 5′-ACC GAC CCC TTC ATT-3′ and 5′-TCC ACG ACA TAC TCA GCA C-3′. As a standard for the copy number of the PCR products, serial concentrations of each PCR fragment were amplified in the same manner. The amount of prepro-ET-1 was calculated as the copy number of each reverse-transcription product equivalent to total RNA and normalized by the value for G3PDH.

### Measurement of ET-1 peptide levels by ELISA

The cerebrum in sagittal brain sections was homogenized in 200 µL of the lysis buffer as described above. The lysates were centrifuged at 15,000 g for 10 min. The amount of mouse ET-1 in the supernatant was measured using an ELISA kit against mouse ET-1 (Phoenix Pharmaceuticals Inc., Burlingame, CA, USA) according to the manufacturer’s protocol. The protein content was measured by BCA protein assay. Data are shown as calculated by ET-1 content per mg of protein in the cerebrum.

### Immunohistochemistry for GFAP

After cold injury, anesthetized mice were perfused from the left cardiac ventricle with 50 mL of PBS followed by 50 mL of 4% paraformaldehyde. Isolated brains were fixed in 4% paraformaldehyde overnight and then soaked in 15% sucrose overnight at 4°C. The fixed brain was placed on a brain slicer and a sagittal section (6 mm thick, between 0 and 6 mm posterior to bregma) was cut. These slices contained a “core” region of injured cerebrum about 0–3 mm from bregma, which was characterized by hemorrhage under the meninges. The slices also contained the cerebral cortex surrounding the core region (4–6 mm from bregma), which we defined as the “peri-core region” of the cold injury. Six frozen sections (30-µm thickness) of the cerebral cortex were obtained from the peri-core region at intervals of about 240 µm, and mounted on slide glasses. After antigen retrieval by soaking specimens in 10 mM citric acid buffer (pH 8.5) for 30 min at 80–85°C, sections were treated with goat serum containing 0.1% Triton X-100 for 1 h at room temperature. Sections were incubated with a monoclonal antibody against GFAP (clone GA5; Sigma-Aldrich, St. Louis, MO, USA) overnight at 4°C. GFAP-positive cells were labeled with a rhodamine-conjugated anti-mouse IgG antibody (Millipore, Temecula, CA, USA) and observed under an epifluorescence microscope. GFAP-positive cells in the cerebral cortex were counted in each of six sections. Results are indicated as the number of GFAP-positive cells per mm^2^ of observed area.

### Statistical analysis

The groups of sham (n = 5–9), vehicle (n = 5–9) and ET antagonists (n = 5–9) were compared. Data were expressed as mean ± SEM. Statistical significance of differences between groups was analyzed by one-way analysis of variance (ANOVA) followed by Dunnett’s test or Tukey’s test.

## Results

### Characterization of edema formation and expression of ET-1 after cold injury in mouse cerebrum

**Figure 2:**
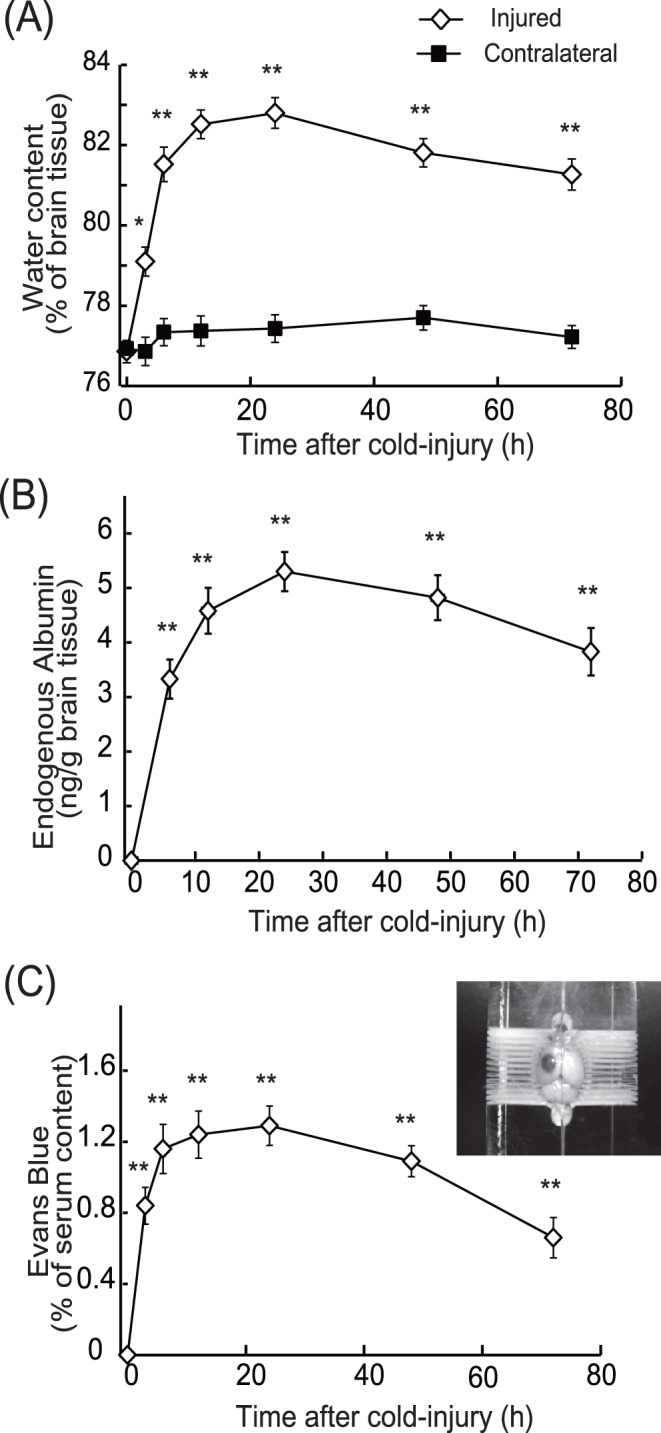
Increases in brain water content and disruption of BBB after cold injury. **(A): Time-course of increases in brain water content.** After cold injury in the left hemisphere of mouse brain, 4-mm sagittal brain sections containing the injured area were made at the times indicated. Water content was calculated by the formula: water content (%)  =  (wet weight−dry weight)×100/wet weight. Results are means ± SEM of six to eight experiments. *p<0.05, **p<0.01 vs. 0 h by one-way ANOVA followed by the Dunnett’s test. **(B) Time-courses of extravasation of endogenous albumin:** After cold injury, mice were perfused with 50 mL saline, and the injured hemispheres were removed at the times indicated. Endogenous albumin in the cerebrum was measured using an ELISA kit. Results are means ± SEM of six to eight experiments, expressed as albumin content (ng) per weight of cerebral tissue (g). **p<0.01 vs. 0 h by one-way ANOVA followed by the Dunnett’s test. **(C) Time-courses of extravasation of Evans blue:** After cold injury, Evans blue was injected into the tail vein 90 min before removal of the injured hemisphere. The amount of Evans blue was measured and expressed as percentage of Evans blue in brain (µg/g) to that of serum (µg/mL). Results are means ± SEM of six to nine experiments. **p<0.01 vs. 0 h by one-way ANOVA followed by the Dunnett’s test. Photograph shows a representative example of Evans blue extravasation 12 h after cold injury.

**Figure 3:**
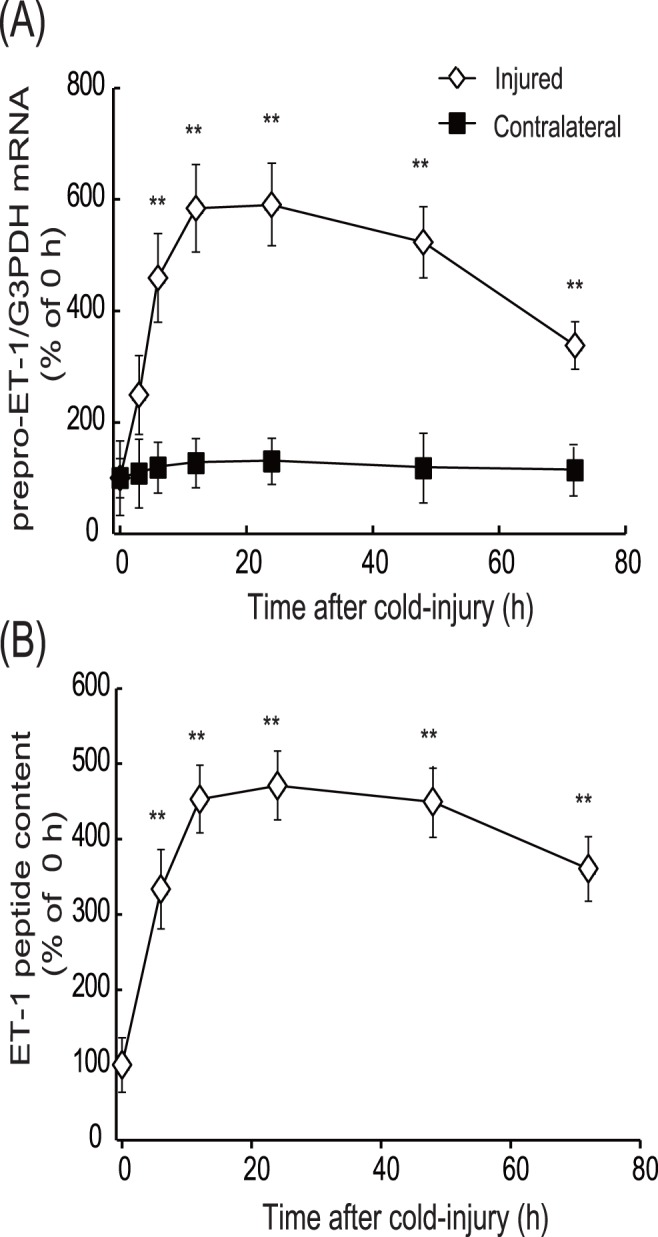
Increases in ET-1 expression after cold injury. **(A) Time-course of increases in prepro-ET-1 mRNA:** After cold injury, expression levels of prepro-ET-1 mRNA in the injured and the contralateral hemispheres were measured at the times indicated. Expression levels of prepro-ET-1 mRNA were normalized to that of G3PDH. Results are means ± SEM of nine experiments. **p<0.01 vs. 0 h by one-way ANOVA followed by the Dunnett’s test. **(B) Time-course of increases in ET-1 peptide:** After cold injury, expression levels of ET-1 peptide in the injured hemispheres were measured at the times indicated. Expression levels of ET-1 peptide in the injured hemisphere were normalized to protein content in the cerebrum. Results are means ± SEM of nine experiments and are expressed as % of 0 h, **p<0.01 vs. 0 h by one-way ANOVA followed by the Dunnett’s test.

Brain cold injury is often used as a model of vasogenic edema, because it increases permeability of brain microvessels by direct actions on vascular endothelial cells [27], [28], [29]. We characterized edema formation and expression of ET-1 after cold injury to mouse cerebrum. Application of a cold copper rod to mouse skull increased water content in the cerebrum of the injured hemisphere (Fig. 2A). Water content in the injured hemisphere was increased to about 83% at 12–24 h, and the increase in water content was sustained 72 h after cold injury. Cold injury did not affect water content of the contralateral hemisphere (Fig. 2A). Extravasation of serum proteins through disrupted brain microvessels underlies development of vasogenic edema. Accumulation of endogenous serum albumin in the injured cerebrum was measured (Fig. 2B). While extravasation of serum albumin was not detected in non-injured brain, cold injury induced accumulation of albumin. The time-course of albumin accumulation correlated with that of water content increase in the injured hemisphere. Disruption of BBB after cold injury was evaluated by extravasation of intravenously injected Evans blue (Fig. 2C). Extravasation of Evans blue in the injury region was observed at 6–72 h after cold injury, although extravasation was not detected in non-injured cerebrum (Fig. 2C). Quantitative PCR analysis indicated that the levels of prepro-ET-1 mRNA in the injury sites were increased at 6–72 h after cold injury (Fig. 3A). Cold injury also increased production of ET-1 peptide in the injured hemisphere (Fig. 3B).

### Effects of ET_B_ antagonists on brain edema formation after cold injury

**Figure 4:**
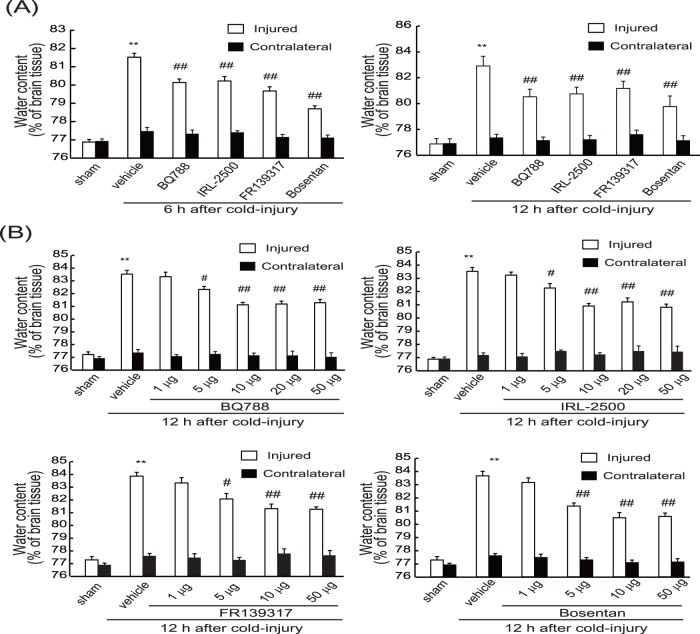
Effects of ET antagonists on brain water content (Protocol 1: Administration prior to cold injury). **(A) Comparison of the effects of ET antagonists.** Ten µg of BQ788, IRL-2500, FR139317, or bosentan was injected into the right cerebroventricle 30 min before cold injury. Mouse brains were removed at 6 h or 12 h after cold injury. Water content in the injured and the contralateral hemisphere was measured. Results are means ± SEM of six to eight experiments. **p<0.01 vs. Sham-operated group, ^##^p<0.01 vs. vehicle administration by one-way ANOVA followed by the Tukey’s test. **(B)** **Dose-dependent effects of ET antagonists.** BQ788, IRL-2500, FR139317 and bosentan at the dosages indicated were administered 30 min before cold injury. Water content in the injured and the contralateral hemisphere was measured at 12 h after cold injury. Results are means ± SEM of five experiments. **p<0.01 vs. Sham-operated group, #p<0.05, ##p<0.01 vs. vehicle administration by one-way ANOVA followed by the Tukey’s test.

**Figure 5:**
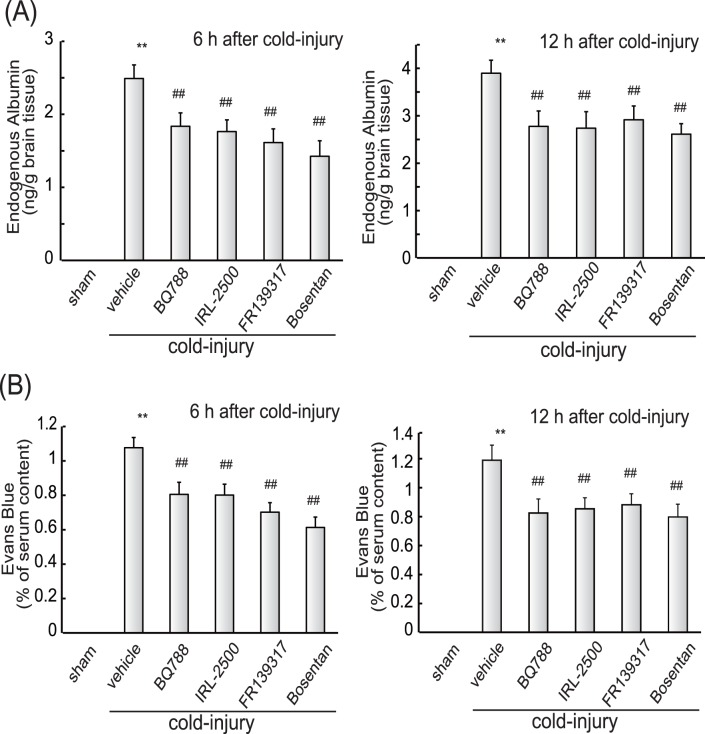
Effects of ET antagonists on extravasation of albumin and Evans blue (Protocol 1). **(A) Endogenous albumin:** Ten µg of BQ788, IRL-2500, FR139317, or bosentan was injected into the right cerebroventricle 30 min before cold injury. Mouse brains were removed at 6 or 12 h after cold injury and albumin content in the injured hemisphere was measured. Results are means ± SEM of six experiments. **p<0.01 vs. Sham-operated group, ^#^p<0.05, ^##^p<0.01 vs. vehicle administration by one-way ANOVA followed by Tukey’s test. **(B) Evans blue:** ET antagonists (10 µg) were injected into the right cerebroventricle 30 min before cold injury. The amount of Evans blue in the injured hemisphere was measured at 6 or 12 h after cold injury. Results are means ± SEM of six to nine experiments. **p<0.01 vs. Sham-operated group, ^#^p<0.05, ^##^p<0.01 vs. vehicle administration by one-way ANOVA followed by Tukey’s test.

Both brain water content and prepro-ET-1 expression were shown to increase concomitantly after cold injury (Figs. 2A and 3), reaching a maximum at 12–24 h, with increased levels persisting at 72 h after injury. Therefore, the involvement of the increased ET-1 in brain edema formation was examined at two different stages, i.e., the early (developmental) phase and the later (persistent) phase. At first, an involvement of ET-1 in the developmental phase was examined by administering ET antagonists prior to the cold injury. When 10 µg of the selective ET_B_ antagonists BQ788 or IRL-2500 was injected in the lateral cerebral ventricle 30 min before cold injury, the increases in water content in the injured hemisphere at 6 and 12 h were significantly reduced (Fig. 4A). The effects of pre-administration of BQ788 or IRL-2500 were smaller at 72 h after cold injury (data not shown). FR139317, a selective ET_A_ antagonist, and bosentan, a non-selective ET antagonist, also decreased the water content of the injured hemisphere (Fig. 4A). The effects of BQ788, IRL-2500, FR139317 and bosentan showed dose-dependency and significant effects were observed at the doses over 5 µg (Fig. 4B). Intracerebroventricular administration of these ET antagonists did not affect water content in the contralateral hemisphere (Fig. 4). Intracerebroventricular administration of BQ788 and IRL-2500 prior to cold injury reduced extravasation of endogenous albumin and Evans blue at 6 and 12 h after injury (Fig. 5A and B). FR139317 and bosentan also reduced extravasation of endogenous albumin and Evans blue (Fig. 5A and B).

### Effects of ET_B_ antagonists on persistence of brain edema induced by cold injury

**Figure 6:**
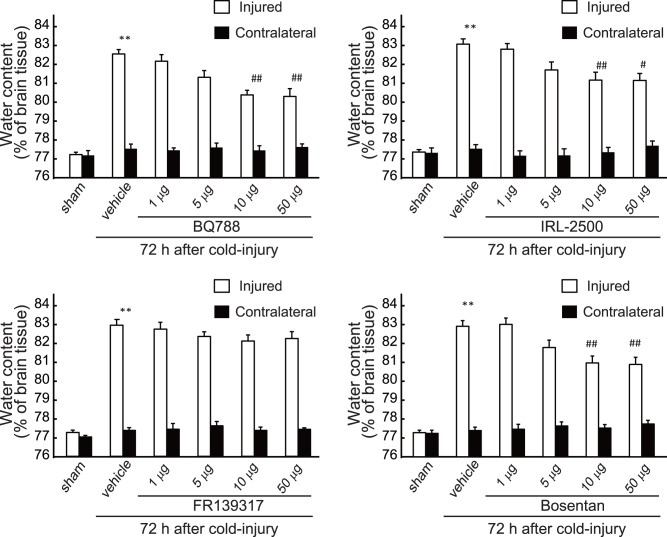
Effects of ET antagonists on brain water content (Protocol 2: Repeated ICV administration after cold injury). BQ788, IRL-2500, FR139317 and bosentan at the dosages indicated were repeatedly administered at 12-h intervals beginning at 24 h after cold injury. Water content in the injured and the contralateral hemisphere was measured at 72 h after cold injury. Results are means ± SEM of five experiments. **p<0.01 vs. Sham-operated group, #p<0.05, ##p<0.01 vs. vehicle administration by one-way ANOVA followed by the Tukey’s test.

**Figure 7:**
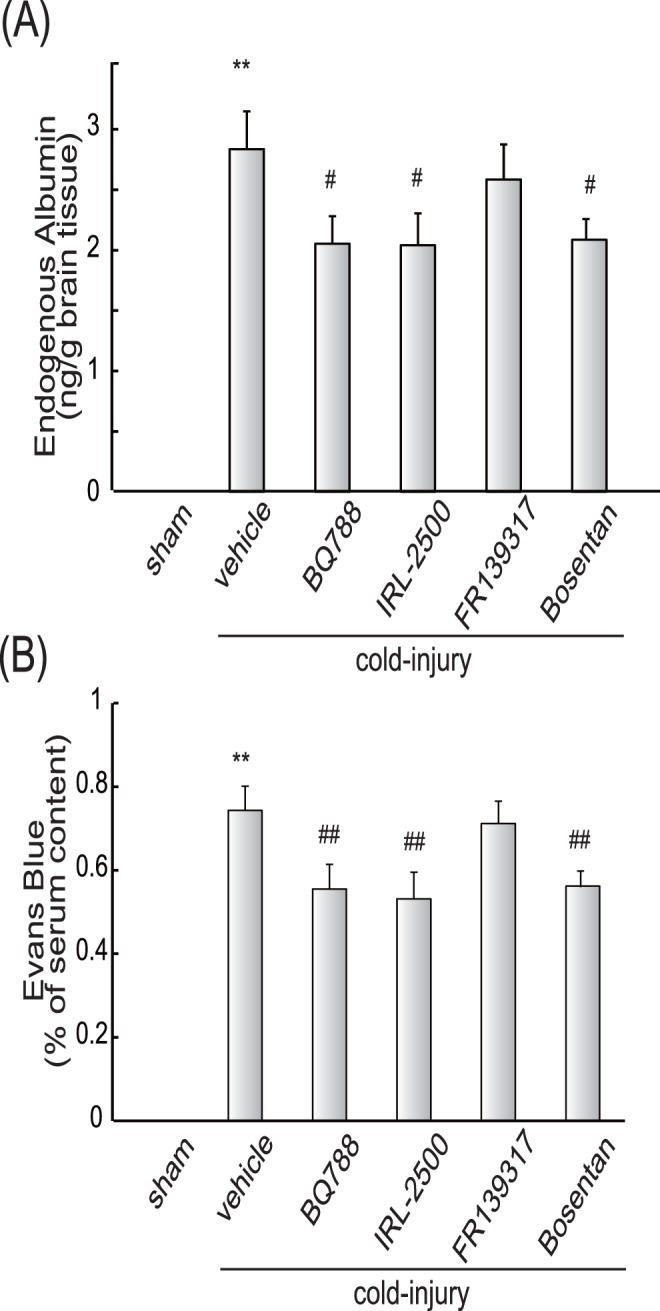
Effects of ET antagonists on extravasations of albumin and Evans blue (Protocol 2: Repeated ICV administration after cold injury). Ten µg of BQ788, IRL-2500, FR139317, or bosentan was repeatedly administered at 12-h intervals beginning at 24 h after cold injury. **(A)** **Endogenous albumin:** Mouse brains were removed at 72 h after cold injury and albumin content in the injured hemisphere was measured. Results are means ± SEM of six experiments. **p<0.01 vs. Sham-operated group, ^#^p<0.05 vs. vehicle administration by one-way ANOVA followed by the Tukey’s test. **(B) Evans blue:** Amount of Evans blue in the injured hemisphere was measured at 72 h after cold injury. Results are means ± SEM of six experiments. **p<0.01 vs. Sham-operated group, ^#^p<0.05, ^##^p<0.01 vs. vehicle administration by one-way ANOVA followed by the Tukey’s test.

Next, the involvement of ET-1 in the persistent phase of brain edema was examined by repeated administration of ET antagonists beginning at 24 h after cold injury. Repeated administration of BQ788 and IRL-2500 after cold injury significantly reduced the water content of the injured hemisphere at 72 h after injury (Fig. 6). Repeated administration of bosentan also reduced the injury-induced increases in water content when administered after cold injury, but FR139317 had no obvious effects even at 50 µg (Fig. 6). Administration of ET antagonists had no effect on water content in the contralateral hemisphere. Administration of BQ788, IRL-2500, or bosentan after cold injury decreased extravasation of endogenous albumin and Evans blue in the injured hemisphere (Fig. 7A and B). Conversely, FR139317 did not affect extravasation of endogenous albumin or Evans blue.

### Effects of ET_B_ antagonists on induction of reactive astrocytes after cold injury

**Figure 8:**
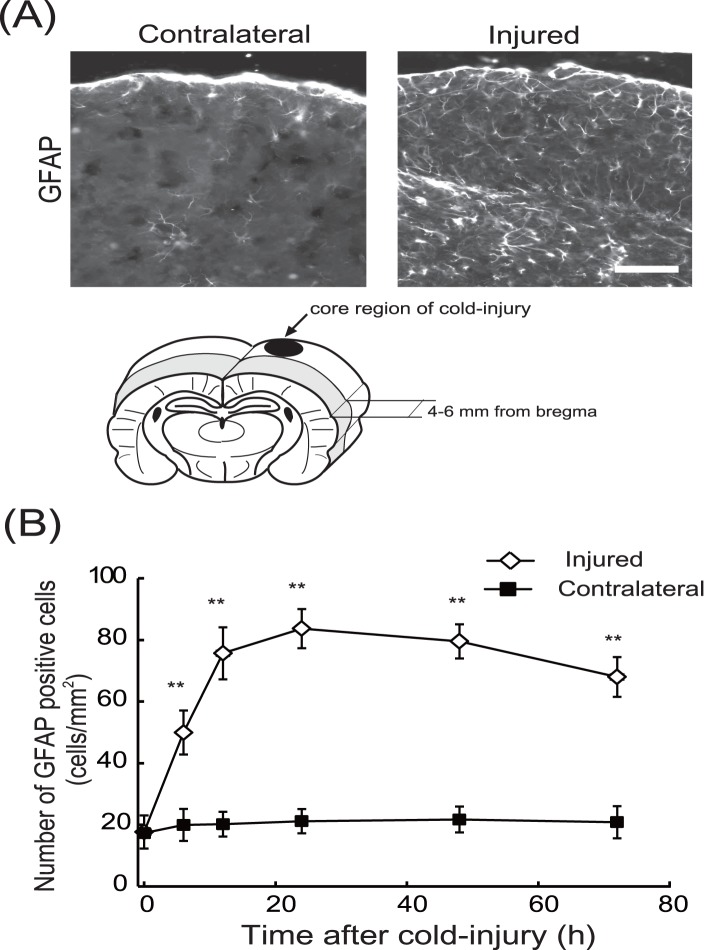
Induction of reactive astrocytes after cold injury in mouse cerebrum. (**A**) Representative photographs showing GFAP-positive reactive astrocytes in the injured and contralateral areas 12 h after cold injury. Induction of reactive astrocytes was observed in sagittal brain sections positioned 4–6 mm from bregma. These sections contained peri-core regions around the core areas damaged by cold injury. Scale bar = 200 µm. (**B**) Time-course of the increase in GFAP-positive cells in the injured regions. GFAP-positive cells in the injured and the contralateral hemisphere were counted at the times indicated after cold injury. The numbers of GFAP-positive cells are shown as numbers of positive cells per mm^2^ of observed area. Results are means ± SEM of six experiments. **p<0.01 vs. 0 h by one-way ANOVA followed by the Dunnett’s test.

**Figure 9:**
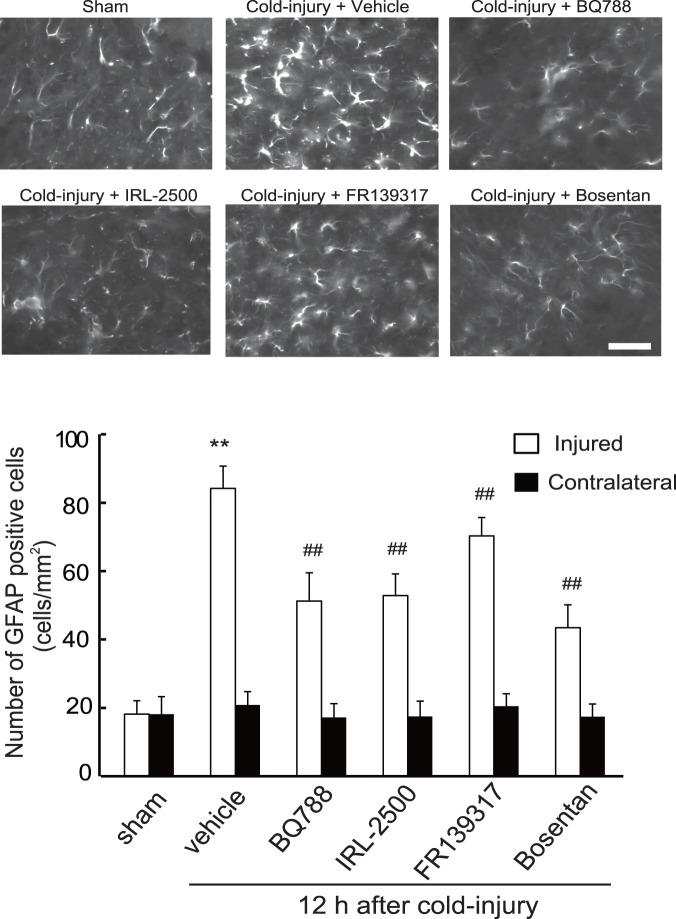
Effects of ET antagonists on induction of GFAP-positive astrocytes by cold injury (Protocol 1). Ten micrograms of BQ788, IRL-2500, FR139317, or bosentan was injected into the right cerebroventricle 30 min before cold injury. Mouse brains were fixed at 12 h after cold injury. GFAP-positive astrocytes in the injured and the contralateral hemisphere were counted. Representative photographs of the injured hemisphere are shown. Scale bar = 50 µm. Results are means ± SEM of six experiments. **p<0.01 vs. Sham-operated group, ^##^p<0.01 vs. vehicle administration by one-way ANOVA followed by the Tukey’s test.

**Figure 10:**
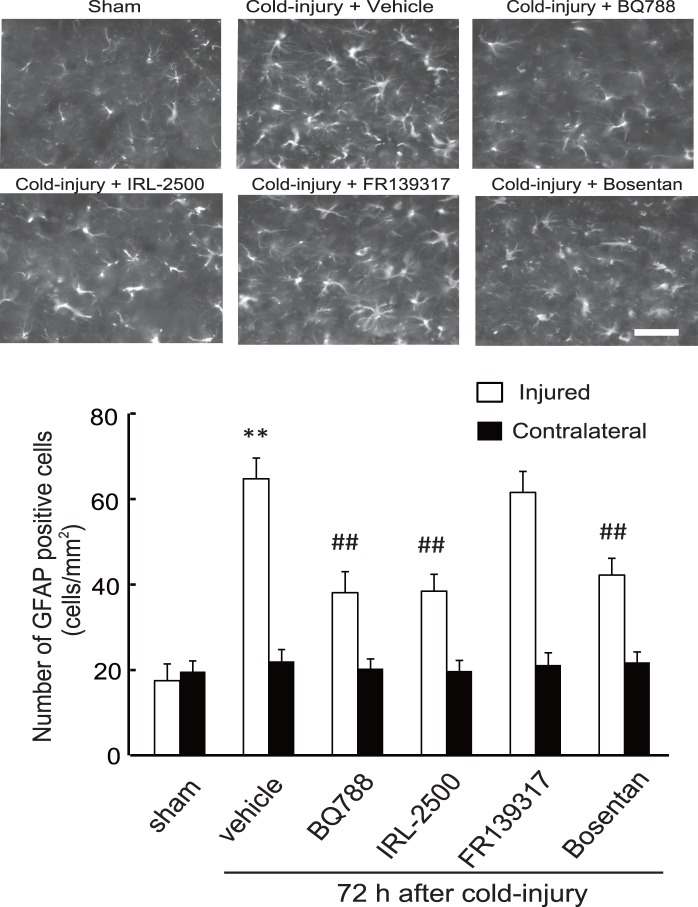
Effects of ET antagonists on induction of GFAP-positive astrocytes by cold injury (Protocol 2). ET antagonists (10 µg) were repeatedly administered at 12-h intervals from 24 h after cold injury. Mouse brains were fixed at 72 h after cold injury. GFAP-positive astrocytes in the injured and the contralateral hemisphere were counted. Representative photographs of the injured hemisphere are shown. Scale bar = 50 µm. Results are means ± SEM of six experiments. **p<0.01 vs. Sham-operated group, ^##^p<0.01 vs. vehicle administration by one-way ANOVA followed by the Tukey’s test.

Resting astrocytes convert their phenotype to reactive astrocytes in response to nerve injury. Because several factors enhancing vascular permeability are produced by reactive astrocytes, induction of reactive astrocytes is thought to promote vasogenic brain edema [30], [31], [32], [33]. In non-injured cerebrum, reactive astrocytes, which were identified by immunochemical staining for GFAP, were observed. Cold injury increased the number of reactive astrocytes around the injured core area; approximately 5-fold increases were observed at 12–24 h and these were maintained at 72 h (Fig. 8). The numbers of GFAP-positive astrocytes in contralateral cerebrum were not altered by cold injury. Administration of BQ788, IRL-2500, or bosentan prior to cold injury decreased the numbers of GFAP-positive astrocytes around the injured area (Fig. 9). The numbers of GFAP-positive astrocytes were also decreased by FR139317, but the degree of decrease was smaller than those observed with ET_B_ antagonists. Repeated administration of BQ788, IRL-2500, and bosentan 24 h after cold injury also decreased the number of GFAP-positive astrocytes in the injured hemisphere (Fig. 10). FR139317 had no significant effect on GFAP-positive astrocytes when administered after cold injury. The numbers of GFAP-positive astrocytes in the contralateral hemisphere were not affected by these ET antagonists, regardless of the administration protocol.

## Discussion

### Vasogenic edema formation and expression of ET-1 in mouse cerebrum after cold injury

Vasogenic edema, which is accompanied by elevation of intracranial pressure, is induced by extravasation of blood proteins through the disrupted BBB [2]. Following stroke and head trauma, production of vascular permeability factors in damaged areas is increased, and excessive actions of these factors reduce the barrier functions of brain microvessels to allow extravasation of blood proteins [34], [35], [36], [37], [38]. Because of the similarity of pathogenesis to brain insults, focal cold injury is used to experimentally induce vasogenic edema. Following brain insults, production of ET-1 is increased and the increased ET-1 promotes various pathophysiological responses in damaged brain [16], [17]. To clarify possible roles of ET-1 in vasogenic edema formation, expression of ET-1 was determined in our mouse cold injury model. As has been reported in previous studies [29], water content and extravasation of markers in mouse cerebrum were increased after cold injury in our model (Fig. 2). The patterns of increases in brain ET-1 after cold injury were correlated with the development of brain edema and disruption of the BBB (Fig. 3). The prolonged elevation of brain ET-1 peptide is consistent with an observation by Hama et al. [39] in a rat cold injury model. The concomitant increases in brain ET-1 production and water content raise the possibility that ET-1 can affect cold injury-induced vasogenic edema in both the development and the persistent phases. Thus, roles of ET-1 in the development and persistence of vasogenic edema were subsequently examined using ET receptor antagonists.

### Differential involvement of ET_A_ and ET_B_ receptors in cold injury-induced vasogenic edema

ET-1 has two distinct receptors, ET_A_ and ET_B_ receptors, which play different roles in the regulation of the pathophysiological responses [18]. In this study, both ET_A_ and ET_B_ antagonists were shown to have inhibitory actions on increases in brain water content in injured hemispheres (Fig. 4). The inhibitory actions of ET antagonists were not caused by stimulating exhaustion of brain water, because water content in the contralateral hemisphere was not affected. ET antagonists also inhibited extravasation of Evans blue and endogenous albumin after cold injury (Fig. 5), so the reduction of brain water content by ET antagonists was most likely due to protection of the BBB. The present observations also produced the notable result that ET_A_ and ET_B_ antagonists had differential inhibitory potencies on vasogenic edema depending on the timing of drug administration. Following administration prior to cold injury, both ET_A_ and ET_B_ antagonists similarly inhibited development of brain edema and disruption of BBB (Figs. 4 and 5). Conversely, FR139317 did not show ameliorating actions following repeated administration from the time when vasogenic edema had been established, although BQ788 and IRL-2500 improved accumulation of water content and disruption of BBB in edematous brain (Fig. 6 and 7). These properties of ET antagonists suggest that the ET_A_- and ET_B_-mediated mechanisms were differentially involved in the cold injury-induced vasogenic edema, with ET_A_-mediated mechanisms involved predominantly in the early developmental phase.

As for inhibitory actions on the development of brain edema, SB234551 and S-0139, ET_A_ selective antagonists, reduced brain edema and disruption of BBB after brain ischemic and traumatic injuries [40], [41]. Together with these studies, our findings with FR139317 indicate a role of ET_A_ receptors in the development of vasogenic edema. Activation of brain ET_A_ receptors that are present in vascular smooth muscle causes vasospasm and results in aggravation of brain ischemia. Therefore, the anti-edema actions of ET_A_ antagonists have been considered to result from reduction of ischemic damage. As for the anti-edema actions of ET_B_ antagonists, few studies have been conducted. However, recent studies by Moldes et al. [42] showed that administration of BQ788 just after middle cerebral artery occlusion potentiated the anti-edema actions of an ET_A_ antagonist, suggesting an involvement of ET_B_-mediated mechanisms in inducing vasogenic edema after brain ischemia. The present study revealed a characteristic property of ET_B_ antagonists, i.e., their anti-edema actions can be obtained when they are administered after vasogenic edema has been established (Fig. 6 and 7). Together with the inhibitory actions on development of brain edema, the ameliorating actions of BQ788 and IRL-2500 on edematous brain suggest that ET_B_-mediated mechanisms work throughout vasogenic edema formation after cold injury.

### Possible involvement of reactive astrocytes in vasogenic edema formation

In brain, ET_B_ receptors are predominantly expressed in astrocytes [19], [20], [21]. Astrocytes surround brain microvessels with the end-feet of their processes. This anatomical link between astrocytes and vascular endothelial cells plays a role in maintaining the functions of the BBB, because the permeability of vascular endothelial cells is modulated by several astrocyte-derived factors. Following brain insults, astrocytes convert their phenotype to reactive astrocytes. Production of astrocyte-derived vascular permeability factors is increased with the astrocytic activation [8], [9]. Excessive action of the astrocyte-derived factors has been thought to be involved in the pathogenesis of BBB disruption leading to brain edema [6], [7]. In fact, recent studies showed that inhibition of astrocytic activation caused amelioration of brain edema, suggesting that induction of reactive astrocytes largely affects brain edema formation [43], [44], [45]. In this study, inhibition of cold injury-induced vasogenic edema by ET_B_ antagonists was accompanied by reduction of reactive astrocytes, irrespective of the timing of drug administration (Figs. 9 and 10). These findings suggest that induction of reactive astrocytes underlies the ET_B_-mediated mechanisms of vasogenic edema formation after cold injury. Because inhibition of vascular permeability factors such as MMP and VEGF ameliorated disruption of the BBB caused by brain ischemia and traumatic injury, the excessive actions of astrocyte-derived vascular permeability factors are thought to have pivotal roles in vasogenic edema formation [10], [11], [12], [13], [14]. In damaged brain, reactive astrocytes become a major source of vascular permeability factors. Activation of ET_B_ receptors stimulated production of astrocyte-derived vascular permeability factors in cultured astrocytes and rat brain [30], [32], [46]. Thus, increased production of vascular permeability factors by reactive astrocytes may be involved in the ET_B_-mediated mechanisms of vasogenic edema formation.

### Pharmacological significance of ET_B_ receptors as a target of neuroprotective drugs

Brain edema is a potentially fatal condition occurring in the acute phases of stroke and head trauma and medications to prevent edema formation have not been established [1], [2], [4], [5]. The present findings that ET_B_ antagonists reduce brain edema and disruption of BBB following cold injury indicate ET_B_-mediated mechanisms of vasogenic edema formation. Beneficial actions of ET_A_ antagonists on brain insults have been examined and ET_A_ receptors are expected to be a promising target of neuroprotective drugs. In addition to ET_A_ receptors, involvement of ET_B_-mediated mechanisms in brain edema formation suggests that ET_B_ receptors could be a possible target of neuroprotective drugs to ameliorate brain damage caused by stroke and head trauma. Recently, Leonard et al. [47] reported that intravenous administration of an ET_B_ agonist ameliorated brain edema and infarct formation in a rat middle cerebral artery occlusion (MCAO) model. This observation apparently contradicts the present findings. However, this conflict could be explained by different mechanisms to induce brain edema between ischemic damage and cold injury. Improvement of blood flow by production of endothelial cell-derived vasodilators was suggested to underlie the beneficial effects of the ET_B_ agonist in the MCAO model. In contrast, improvement of blood flow may not have a large influence on the cold injury-induced brain edema. This idea is supported by the restrictive inhibitory action of an ET_A_ antagonist, which improves blood flow in a damaged brain, on the brain edema. Cold injury cannot fully replicate the pathological aspects of stroke and head trauma, although it is used as a model of brain edema. Therefore, the effects of ET_B_ antagonists are required to be evaluated in other brain injury models to confirm roles of the ET_B_-mediated mechanisms of brain edema formation in brain insults.
